# Intentional incisions on Neolithic Obsidian arrowheads from Central Anatolia: A techno-functional and iconographic approach

**DOI:** 10.1371/journal.pone.0354715

**Published:** 2026-07-28

**Authors:** Alice Vinet, Denis Guilbeau

**Affiliations:** 1 UMR 7269 LAMPEA, Aix Marseille Université, CNRS, Ministère Culture, Aix-en-Provence, France; 2 Institut Français d’Etudes Anatoliennes (IFEA), Istanbul, Türkiye; 3 Ministère de la Culture, Montpellier, France; 4 UMR 5140 ASM, Montpellier, France; Sapienza University of Rome: Universita degli Studi di Roma La Sapienza, ITALY

## Abstract

Incised obsidian arrowheads form a rare and geographically restricted artifact class, known exclusively from Neolithic Central Anatolia between the late 8th and 7th millennia BCE. These artifacts, marked by deliberate incisions, have been variously interpreted as symbolic or identity-related. This study presents a comprehensive techno-functional and iconographic analysis of eleven newly identified specimens from Tepecik-Çiftlik (Cappadocia, Türkiye), adding to the regional corpus of 43 known examples. The incised arrowheads were found in dwelling sites, a chipped stone workshop, and during surveys.Provenance analysis by macroscopic characterization confirms their production from local Cappadocian obsidian sources (Göllüdağ, Nenezi Dağ). The incisions are highly variable, and show no correlation with raw material, *chaîne opératoire*, typology, or use-wear. Incisions were executed both before and after final retouching, suggesting no fixed production stage. Comparative analysis reveals parallels between sites, indicating a possible shared but flexible iconographic repertoire. The rarity of these artifacts, their relation to domestic contexts, and their absence beyond Central Anatolia point to the hypothesis of a shared cultural practice. The analysis of these incised arrowheads, along with the limited archaeological and ethnographic comparisons available, calls for a cautious interpretation. While they have been suggested as personal or collective markers (e.g., hunters’ marks), this hypothesis requires further evidence. The expanded dataset from Tepecik-Çiftlik refines the understanding of this peculiar practice, highlighting its persistence despite broader cultural change.

## Introduction

### Regional settings

**Fig 1:**
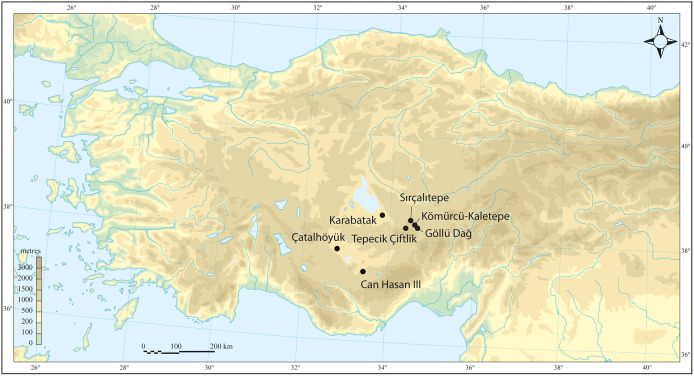
Map showing the location of the sites cited in the text. Base map from M. Sauvage, modified by the authors.

Incised obsidian arrowheads represent a particularly intriguing category of artifacts. These objects, characterised by deliberate incisions, are exceptionally rare in the archaeological record. To date, they have been found only in Central Anatolia, where they are predominantly associated with contexts dating to the late 8th and 7th millennia BCE (Fig 1, Table 1). Their scarcity, combined with the specificity of their incisions, has raised important questions regarding their potential symbolic meanings, social functions, or utilitarian purposes. Whether interpreted as markers of individual or collective identity, or expressions of ritual behaviour, among other hypothesis, incised arrowheads provide a unique window into the symbolic and cultural dimensions of prehistoric communities [1–4].

**Table 1:** Inventory of the incised arrowheads.

Sites	Level	Dates cal BCE	Incised points	References
Can Hasan III	Phase 2; topsoil	7650−6600	31	Ataman 1988
Çatalhöyük	Level South G; Level North H; Level South K; Trench 7	7100−6050	5	Doyle 2021
Göllüdağ Kuşburnu/Kepez (localities 116, 117, 120)	Topsoil	Aceramic Neolithic	1	Balkan-Atlı et al. 2008
Karabatak 11	Topsoil	Late Aceramic Neolithic	2	Erdoğu et al. 2007
Kömürcü-Kaletepe	Layer 1 area P	8300−8200	1	Balkan-Atlı and Binder 2000
Sırçalıtepe		mid-8th mil.	2	Balcı et al. 2022
Tepecik-Çiftlik	level 10–3; topsoil	7100−5800	12	This paper, Balcı 2019
		***Total***	***54***	

A total of 54 incised arrowheads has been recorded so far. At Can Hasan III, a Neolithic settlement, 31 incised arrowheads were recovered [1]. Only four artefacts with incisions were found in stratified contexts, all from Phase 2, dated between 7650 and 6600 BCE. At Çatalhöyük, a large Neolithic settlement with a long stratigraphic sequence spanning over a millennium and a half, five incised obsidian arrowheads have been documented across different levels dated between 7100 and 6050 BCE [4]. Additional finds include two incised arrowheads discovered during surface surveys at Karabatak 11, where concentrations of artifacts are related to different activities, possibly including salt exploitation. These finds are attributed to the Late Aceramic Neolithic [5]. One additional incised arrowhead was found at Göllüdağ Kuşburnu/Kepez (localities 116, 117, 120), a probable seasonal camp site near rock shelters dedicated to the extraction of raw material and/or hunting during the Aceramic Neolithic [6]. Furthermore, a single specimen was identified at Kömürcü-Kaletepe, an Aceramic Neolithic obsidian workshop, within a layer dated approximately to 8300−8200 BCE [7]. Two incised arrowheads have also been reported from Sırçalıtepe, an Aceramic Neolithic settlement and obsidian workshop [2]. At Tepecik-Çiftlik, occupied between 7100 and 5800 BCE [8], one incised arrowhead was previously published [9], and 11 more were identified and will be described in greater detail in the “Material” section of this paper.

Previous interpretations of incised obsidian arrowheads have primarily focused on the “hunter marks” hypothesis. Ataman [1], based on her work at Can Hasan III, proposed that the incised arrowheads were used as projectiles and that the incisions were likely related to individuals rather than broader community groups. The incised patterns may have had functional purposes associated with hunting activities, identifying the maker and/or owner of the tool, and/or the hunter. To support this interpretation, Ataman referred to ethnographic parallels, notably examples from Inuit groups, where arrows were intentionally marked by individual hunters to allow them to claim ownership of game animals [10–12]. Such ethnographic analogies could suggest that the incisions on the Can Hasan III projectiles could similarly have served to assert individual ownership or hunting skills.

However, this hypothesis faces notable limitations. The apparently random distribution of incisions on the artifacts, combined with the overall scarcity of incised arrowheads across sites, complicates functional interpretations. The random distribution of the artifacts within the sites and the lack of consistent stylistic patterns among the incisions raise questions about the coherence of the phenomenon.

In light of these issues, several fundamental research questions arise: Why are these incised arrowheads so rare in the archaeological record? Which tools and techniques were used to produce them? Did the incisions serve primarily symbolic, technical, or social functions? Addressing these questions, requires a renewed analytical approach is necessary. Since Ataman’s initial study, no comprehensive technological or functional analysis has been carried out on assemblages of incised arrowheads. Furthermore, the corpus of incised projectiles has grown significantly with new finds from Çatalhöyük, Karabatak, Göllüdağ, Kömürcü-Kaletepe, Sırçalıtepe, and Tepecik-Çiftlik. Based on the 11 new finds at Tepecik-Çiftlik, a thorough examination of the artifacts from this site is crucial to determine whether they represent a coherent phenomenon, potentially indicative of specific productions, exchange networks, or symbolic practices, or whether the incisions reflect a more heterogeneous range of behaviours. Comparative analysis of specimens from multiple contexts is essential to fully understand the significance of these incised patterns within the broader framework of Neolithic Central Anatolia.

### Tepecik-Çiftlik

Tepecik-Çiftlik is a long-inhabited, multi-period archaeological settlement located on the Melendiz plain in the volcanic Cappadocia region of Central Anatolia (Niğde Province, Türkiye), at an elevation of approximately 1600 m above sea level. The site forms an oval-shaped mound measuring c. 300 m in length and 170 m in width, rising up to 9.6 m above the surrounding plain. Excavations, conducted since 2000 by a team from Istanbul University, have revealed a long stratigraphic sequence spanning from the late Pre-Pottery Neolithic (Level 10) to the Early Chalcolithic (Level 2), although the bedrock has not yet been reached. The Pottery Neolithic phase (Levels 9–3) is the most intensively studied occupation, dated between approximately 6800 and 6100 cal BCE [8,13,14]. Levels 5–2 have yielded the most substantial information regarding settlement organization and building practices. The site is characterized by domestic structures, including buildings, hearths, and storage features. The remains uncovered provide evidence of agricultural practices within a fully agropastoral economy, involving the exploitation of various domesticated plant species, especially cereals, and the husbandry of different animal species, notably ovicaprines [13]. Hunting of various species was practiced throughout the occupation; however, among the faunal remains, wild species are marginal, except during the Chalcolithic period [15]. To date, more than 100 graves have been identified and excavated at the site, including collective burials and plastered skulls, attesting to a wide range of mortuary practices and treatments of the dead, although human remains have so far only been recovered from Levels 5–3 [16,17]. The site lies in close proximity to the main obsidian outcrops of Cappadocia, Göllüdağ is located 5 km away and Nenezi Dağ approximately 20 km away. These obsidian sources, intensively exploited since the Palaeolithic, likely played a decisive role in the long-term occupation of the site despite the environmental constraints associated with high-altitude farming and herding [9,18]. Obsidian is extremely abundant in each archaeological level, and evidence indicates that multiple *chaînes opératoires*, for both flake and blade production, were at least partly carried out on site [18–21]. The continuity of occupation highlights Tepecik-Çiftlik as a key example of long-term settlement resilience during the Neolithic in Cappadocia.

## Materials and methods

### Inclusivity in global research

The cultural material examined in this paper is stored in one of the storage units at Tepecik-Çiftlik (Niğde Province, Türkiye). The material was collected during the ongoing excavations, incised arrowheads were collected in the 2004, 2009, 2010, 2014 and 2019. Permission for these excavations was granted to Prof. E. Bıçakçı, the late former director of the Tepecik-Çiftlik excavation, and now to Dr. Y. G. Çakan, by the General Directorate for Cultural Heritage and Museums and the Ministry of Culture and Tourism of the Republic of Türkiye. Permission to study the artefacts was granted to the authors by the Ministry of Culture and Tourism of the Republic of Türkiye. Additional information regarding the ethical, cultural, and scientific considerations specific to inclusivity in global research is provided in the Supporting Information (S1 File).

### Material

Incisions were identified on twelve of the 740 arrowheads documented to date, representing 1.6% of the total assemblage (Table 2, S2 File). However, recognising such incisions is only possible if the mesial portion of the artefacts is preserved and if the retouch on the ventral face is not extensive or covering. On retouched surfaces, the arises of flake scars considerably hinder the visibility of incisions, hence the need for flat, unretouched surfaces to make incisions. When considering only the arrowheads with a preserved central section and minimal or no ventral retouching, the proportion bearing incisions increases to approximately 3%. Of the twelve incised arrowheads, one was recovered from the surface, another from a cleaning unit, while three originate from the infill layers of level 3, dated to 6400–6100 BCE and associated with the Pottery Neolithic. Six specimens were found in infill layers of levels 4–6, which date to 6800–6400 BCE, also within the Pottery Neolithic, and one arrowhead was retrieved from infill layer of level 10, dated to 7100–7000 BCE, corresponding to the Pre-Pottery Neolithic [8]. Incised arrowheads are present throughout most of the stratigraphic sequence. They were not identified in levels 7–9; however, this absence can likely be attributed to the limited extent of excavation in these layers. In contrast, no incised arrowheads were found in the Chalcolithic levels. Given the high number of arrowheads recorded across all levels, this absence appears potentially meaningful. Nevertheless, caution is warranted due to the small number of incised specimens identified overall.

**Table 2:** Inventory of the incised arrowheads from Tepecik-Çiftlik.

N°	Year	Square	Unit	Small find	Level	Dates cal. BCE	Length in mm	Width in mm	Thickness in mm	Provenance
1	2004	16K			10	7100−7000	48.8	18.2	5.4	GD type 6
2	2009	17J	151		6−5	6800−6600	46.4	20.7	10.4	ND type 7
3	2009	16K	212		6−5	6800−6600	71.2	24.7	9	ND type 7
4	2010	16K	258	588	6−5	6800−6600	53.2	14.1	5.6	GD type 3
5	2010	18J	174	351	5	6800−6600	66.4	25	8.8	ND type 7
6	2009	18J	98		5−4	6800−6400	65	25.7	10.5	ND type 5
7	2009	18K	109		4	6600−6400	57.5	20.3	7.2	GD type 6
8	2019	17L 18L	192		3	6400−6100	59.7	18.8	6.4	GD type 6
9	2010	18L		88	3	6400−6100	54	17.6	5.3	ND type 10
10	2010	18L	79	170	3	6400−6100	67.8	16.8	8	ND type 5
11	2010	16M	1	12	Surface	/	83.7	21	10.6	GD type 1
12	2014	15K	196		Cleaning	/	35.8	11	5.1	GD type 6

### Methods

The provenance of obsidian was investigated through visual characterization with the naked eye following the method initially developed by N. Kayacan, T. Carter, and M. Milić [3,22,23]. Although no formal provenance analyses were conducted for this assemblage, the macroscopic characterization approach provides a reliable framework for distinguishing obsidian sources. This method relies on a series of visual criteria, including colour, the presence and nature of inclusions, and degrees of transparency, which have been shown to correlate with specific geological sources of obsidian.

This study employs a techno-functional approach that integrates both the analysis of knapping scars and use-wear traces. The technological component of the analysis allows identification of manufacturing techniques through examination of the knapping scars and retouch [24,25]. For the use-wear analysis, a low-power approach was employed [26,27] using a Leica stereoscopic microscope EZ4W (magnifications from 8x to 35x). In this study, the low-power approach is sufficient to identify and describe the incisions, given the nature of the research questions, higher magnification would not provide additional reliable information regarding the production of the incisions. To determine whether the incisions were of anthropogenic origin, three main criteria were established: first, the incision must be visible to the naked eye; second, it must involve the removal of material, rather than being a superficial scratch; and third, the incision must display clear evidence of deliberate execution. This latter criterion is met by the observation of repeated incisions, often double or triple, indicating deliberate and controlled repeated gestures. Despite the generally abraded condition of the surfaces, particularly with frequent post-depositional scratching, the use of low-angle lighting proved highly effective in enhancing the visibility of the incisions, allowing detailed observation and documentation of the incised features (S3 File).

### Experiments

**Fig 2:**
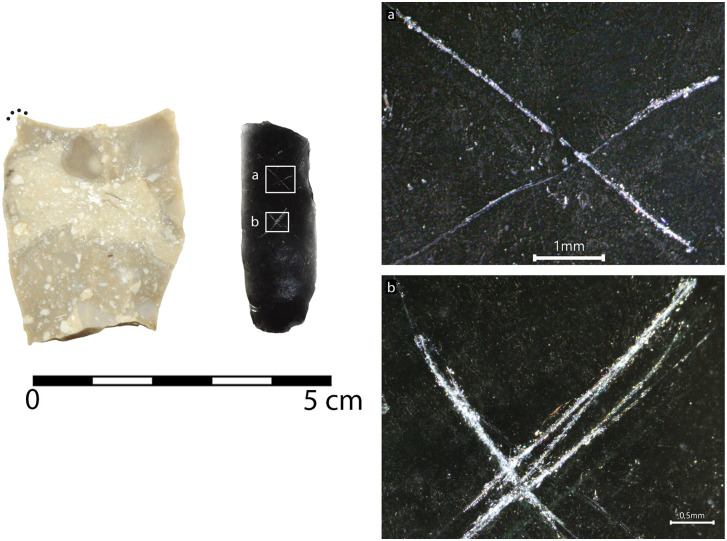
Left: flint tool used to make the incisions, the dots indicate the used area; middle: obsidian blade with crossed incisions; right: details of the incisions.

To better understand the techniques used to produce the incisions observed on the archaeological arrowheads, an experimental replication was carried out. Two crossed incisions were made on the unretouched ventral surface of an obsidian bladelet from Göllüdağ (Fig 2). The incisions were executed using the proximal end of an unretouched flint blade from Gargano (Apulia, Italy). The experiment showed that flint is highly effective in producing precise and well-defined incisions on the obsidian surface. In contrast, attempts to incise obsidian with another obsidian tool were unsuccessful, confirming that a harder material is required to incise obsidian effectively. This observation is consistent with Mohs hardness values: obsidian typically ranges from 5.0 to 5.5, while flint is significantly harder, with a hardness of approximately 7.0. Macroscopic analysis of the experimentally incised surfaces revealed use-wear patterns comparable to those observed on the archaeological artefacts, supporting the hypothesis that a flint tool could have been used to incise the arrowheads. However, at this stage, it is not possible to further specify the exact type of tool used. Fewer than 1% of the chipped stone artefacts at the site are made from raw materials other than obsidian [18–21]. These include a limited number of flakes made on coarse volcanic rocks with poor flaking properties, as well as artefacts made from a light-brown, semi-translucent flint of unknown provenance. The flint assemblage consists primarily of flakes and blades which could have been used to produce the incisions.

## Results

### Chipped stone assemblage of Tepecik-Çiftlik: general settings

Since the beginning of the site’s excavation in the 2000s, several hundred thousand chipped stones have been collected. The first study was conducted by S. Balcı on a sample from a deep sounding corresponding to the oldest levels reached so far [9]. Systematic registration of the chipped stones began in 2016. The composition of the assemblage does not show significant change over time when considering the raw materials, *chaînes opératoires,* and toolkit [18–21,28,29]. More than 99% of the assemblage is made of obsidian, coming from the outcrops of Göllüdağ and Nenezi Dağ. The industry is characterized by the abundance of waste and debris, related to the production of flakes by direct percussion with a hard stone hammer. Unretouched flakes and flake cores are common. Among the waste, the frequency of shaping flakes is striking. These are related to the preparation of blade cores and/or preforms, but cores and preforms are almost absent. Unidirectional blades, produced mostly by direct percussion with a soft stone hammer, are well attested. They are quite irregular, and their manufacture corresponds to a low-technical level of production. In contrast, bidirectional blades, which are rarer, are associated with highly skilled production, involving well-mastered core shaping to produce predetermined blades with a triangular thick section and converging edges. The cores related to this production are entirely absent. The morphology of bidirectional blades was perfectly suited to their main use as arrowhead blanks and required little retouching to be used as such.

Besides scrapers, arrowheads are the most frequent formal tools in the industry, with 740 registered so far. Ten arrowheads were made of flint and 730 of obsidian. Based on visual characterisation with the naked eye, about 56% of the obsidian arrowheads were made on Göllüdağ obsidian, and about 44% on Nenezi Dağ obsidian. Most of these arrowheads are broken, and 144 correspond to small fragments, mainly tips and tangs. The complete or nearly complete arrowheads are about 60–80 mm long and about 20 mm wide. The extent of the retouch prevents detailed characterisation of the blank for 55% of the arrowheads (408 out of 740). Among the 332 clearly identifiable artefacts, 279 (84%) were produced on blades, while 53 (16%) were made on flakes or small blocks. For the 408 arrowheads whose blanks cannot be identified due to retouch, the proportions of arrowheads made on blades and those made on flakes or small blocks are likely to be very similar. Indeed, these 408 arrowheads show no significant dimensional or morphological differences compared with those for which the blank is identifiable. Typologically, different types are present, but they cannot be grouped into clearly distinct categories. For example, arrowheads with pronounced barbs coexist with arrowheads without barbs, and between these extremes are arrowheads with more or less developed barbs. This observation holds for all other criteria of typological variability, such as the proportions of the different parts of the arrowheads and their overall morphology. Most points are tanged and elongated. In some cases, the tang can hardly be distinguished from the mesial part. In many cases, these arrowheads are characterized by an elongated diamond or oval shape. The retouch was done by pressure. The extent varies greatly, some are very lightly retouched, as they are produced on thick blades with a triangular cross-section and convergent edges, and therefore require little or virtually no retouch to be used as arrowheads [19: Fig 3.13]. In other cases, the retouch covers the entire upper and lower faces, without any close relation to the morphology of the blank. The extent of retouch is not correlated with the regularity of the blank. Thus, points made on less regular blanks do not exhibit more extensive retouch than those produced on highly regular, naturally pointed blanks. Among the 596 arrowheads with their mesial part preserved, the lower face of 189 (31.7%) shows a covering retouch. The regularity of the arrowhead shape and retouch also vary. About 50% of these arrowheads are not well-shaped, with an axial dissymmetry, irregular edges and irregular retouch. Conversely, about 25% are characterized by a perfectly symmetrical shape with regular edges and parallel pressure retouch.

### Provenance of incised arrowheads

Six of the 12 incised arrowheads are made from obsidian sourced from Nenezi Dağ and six from the eastern flank of Göllüdağ. Among the Göllüdağ obsidian arrowheads, four correspond to macroscopic type 6, characterized by complete transparency; one to type 3, defined by its transparent matrix with white striations; and one to type 1, a transparent obsidian with grey inclusions. The Nenezi Dağ group includes three pieces of type 7 obsidian, identifiable by its ashy greenish-grey appearance; two of type 5, marked by a greenish-grey colour with darker internal stripes; and one of type 10, which presents an ashy matrix with dark stains.

### Technology of incised arrowheads

At least six arrowheads were made on blades, of which four on bidirectional blades. None were made on unipolar blades or on flakes. The shortest, which is almost complete, is 35.5 mm long (Fig 15d) and the longest is 83.2 mm long, though the distal part is missing (Fig 15c). Their width ranges from 10.8 to 28 mm. Only two are complete (Fig 15d and e), including one that was rejuvenated after a break (Fig 15e). Their morphology, especially their measurements vary, but all correspond to tanged arrowheads. Three are characterized by a very regular shape and regular pressure retouch (Fig 15a, g and l), while three are characterized by an irregular shape and retouch (Fig 15b, c and e). The technological traits of incised arrowheads do not differ from those of the non-incised ones.

### Microscopic analysis of incised arrowheads and characterization of the incisions

This section provides a detailed description of each incised arrowhead. Each incision discussed meets the criteria established in the methodology section, they are visible to the naked eye, involve a clear removal of material, and demonstrate intentionality, except for two arrowheads which will be discussed at the end of this section.

**Fig 3:**
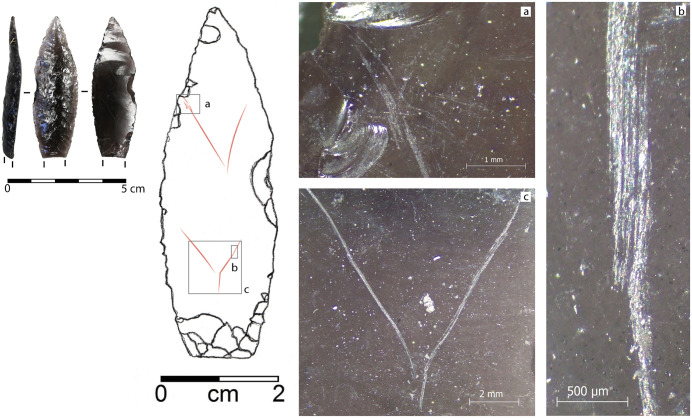
Arrowhead #8 with incisions composed of two repeated V-shaped lines on the mesial section of the ventral face.

Arrowhead #8 bears incisions composed of two repeated V-shaped lines, clearly visible to the naked eye. The incisions are located on the mesial section of the ventral face of the artefact. Although the overall design appears simple at first glance, closer examination reveals that at least nine incisions were required to create the composition. Several of the incised lines are doubled or even tripled (Fig 3A–C), which reinforces their intentional nature. The complexity and repetition of the incisions demonstrate that it was deliberately designed rather than resulting from unintentional scratching. The sequence of the incisions in relation to the retouch remains uncertain. No direct overlap between the incisions and the scars is observable, making it plausible that the incisions were made before, during, or after the retouch process. The arrowhead is complete and does not exhibit any diagnostic impact traces that would indicate its use as a projectile.

**Fig 4:**
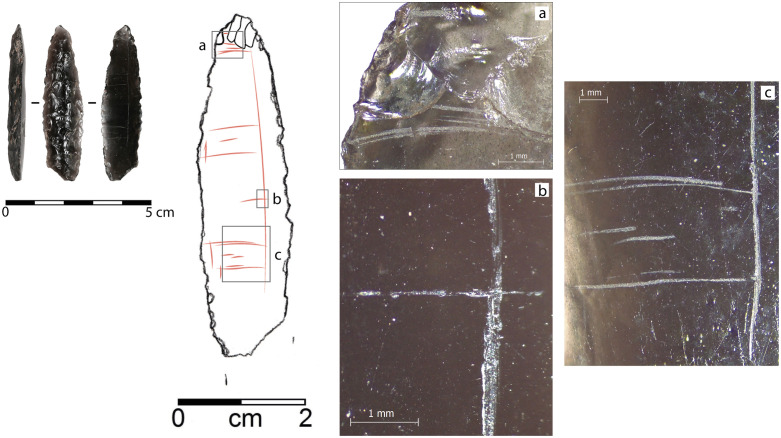
Arrowhead #4 with three repeated square incisions covering the ventral face.

Arrowhead #4 displays complex incisions composed of three repeated square-shaped groups, clearly visible to the naked eye. The incisions extend across the entire ventral face of the artefact. Multiple incisions, some appearing doubled or tripled (Fig 4A-C), were required to produce each square, reflecting a deliberate and careful execution. The repetition and number of overlapping strokes confirm that the incisions were not produced randomly but intentionally composed. This is the most elaborate group of incisions identified within the assemblage, requiring twenty individual incisions. The sequence suggests structure: the three squares were incised first, followed by the addition of a vertical line (Fig 4C). This layering may imply a degree of premeditation in the design process before execution. The incisions were made prior to the inverse retouch, as evidenced by some retouch scars visibly removing parts of the incised lines (Fig 4A). The arrowhead is broken and lacks diagnostic impact fracture.

**Fig 5:**
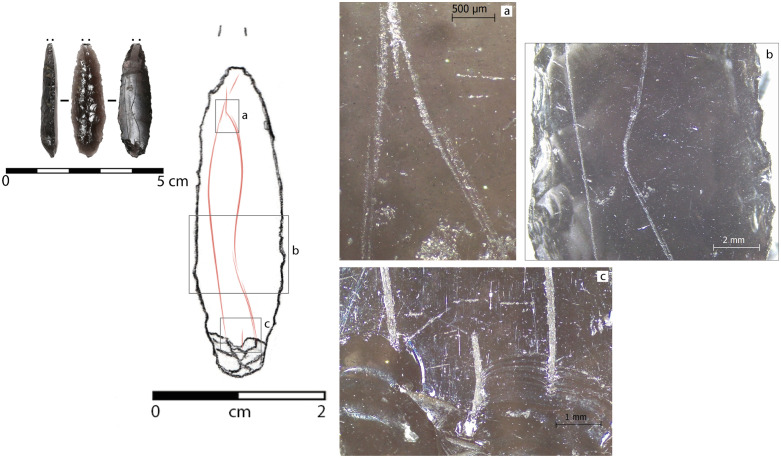
Arrowhead #12 with two converging wavy lines incised across the ventral face.

Arrowhead #12 features a motif of two wavy lines that converge near the distal end of the artefact. The incisions extend across the entire ventral face and are composed of nine individual strokes. Several lines are doubled, reinforcing the intentional nature of the design (Fig 5A-B). The incisions were made after the inverse retouch, as shown by several incised lines that clearly overlap flake scars, particularly in the proximal part (Fig 5C). The arrowhead is fractured in the distal part, but it is not diagnostic and cannot be associated with impact.

**Fig 6:**
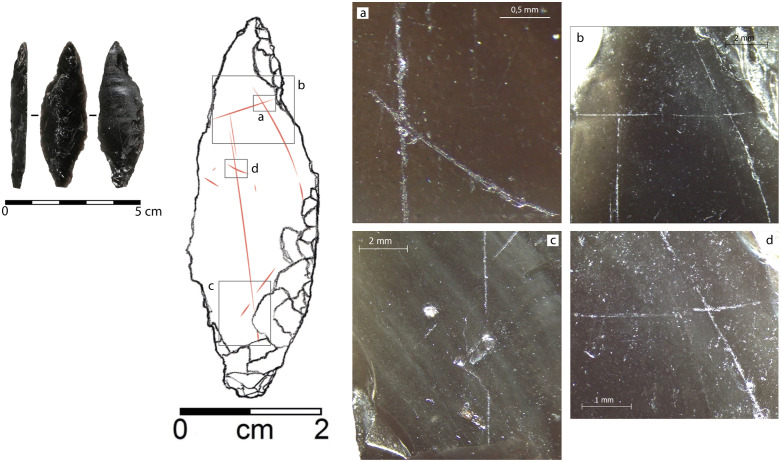
Arrowhead #9 with large roughly rectangular incisions on the ventral face.

Arrowhead #9 bears incisions forming a large, roughly rectangular shape, clearly visible to the naked eye. The incisions are distributed across the entire ventral face of the artefact and are composed of nine individual lines. One vertical line on the left side is doubled (see Fig 6B), further confirming the deliberate nature of the design. The intersections of vertical and horizontal lines show a consistent order of execution: in both observed cases, the vertical incisions were made before the horizontal ones (Fig 6A-D), which implies a planned sequence and supports the hypothesis of intentional structuring. Part of the left vertical incision was subsequently removed by inverse retouch (Fig 6C). This suggests that the incisions predate at least some of the retouch work, although the overall relationship between the two may be more complex. The arrowhead is complete and shows no impact fracture diagnostic of use as a projectile.

**Fig 7:**
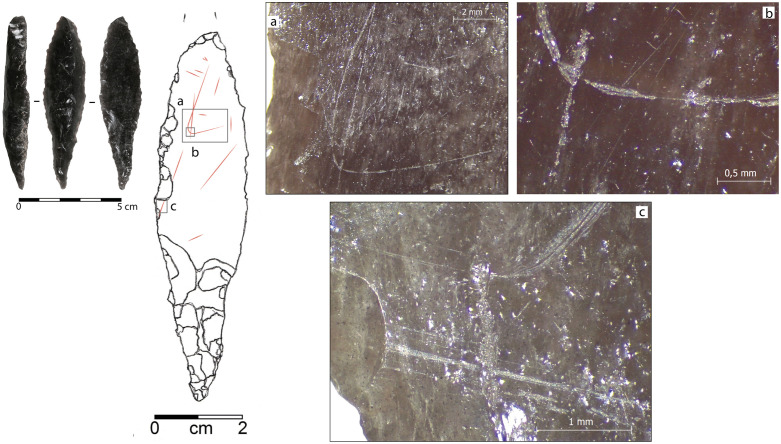
Arrowhead #11 with incisions forming a faint L-shape and additional lines concentrated on the distal half of the ventral face.

Arrowhead #11 exhibits relatively less visible incisions, though they remain discernible to the naked eye. The reduced visibility is partly due to the greyish colour and natural stripes within the raw material, which visually interfere with the incised lines (Fig 7A). The incisions form a somewhat irregular L-shaped design surrounded by other vertical and horizontal lines. Most incisions are concentrated in the distal half of the ventral face. Eleven incisions have been identified, none of which appear to be doubled. One intersection has been documented where the L-shaped incision was made prior to a vertical line that crosses over it (Fig 7B). Part of a vertical incision on the left was removed by later inverse retouch (Fig 7C). The arrowhead displays a large, burin-like impact fracture at the distal end (Fig 7, profile), extending over several centimetres. This fracture is diagnostic and can be associated with projectile use.

**Fig 8:**
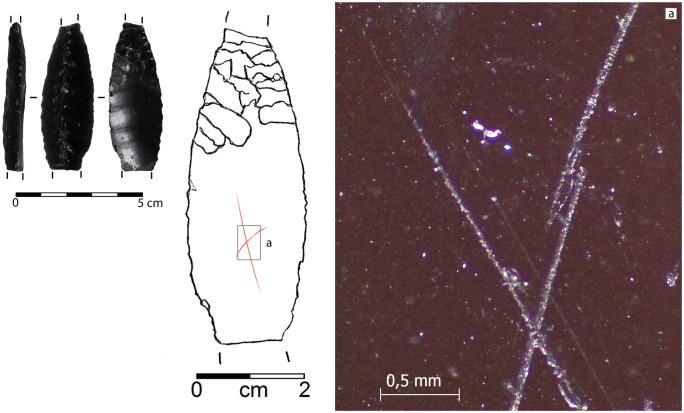
Arrowhead #7 with slightly asymmetrical cross-shaped incisions on the ventral face.

Arrowhead #7 displays incisions forming a cross, clearly visible to the naked eye. The incisions are slightly asymmetrical, with one intersecting line longer than the other. The right incision is doubled on a small section (Fig 8A), indicating the intentional nature of the markings probably intended to extend the line. The cross is centrally positioned on the ventral face, a placement that may have been chosen for its symmetry. Three incisions were required to create the cross. The sequence of incisions appears deliberate: the two right-hand lines were made first, followed by the intersecting line on the left. The chronological relationship between the incisions and the retouch remains unclear. There is no observable overlap between the incisions and the scars, leaving open the possibility that the incisions were made before, during, or after the retouching process. The arrowhead shows fractures at both the distal and proximal ends; however, none are diagnostic of projectile impact.

**Fig 9:**
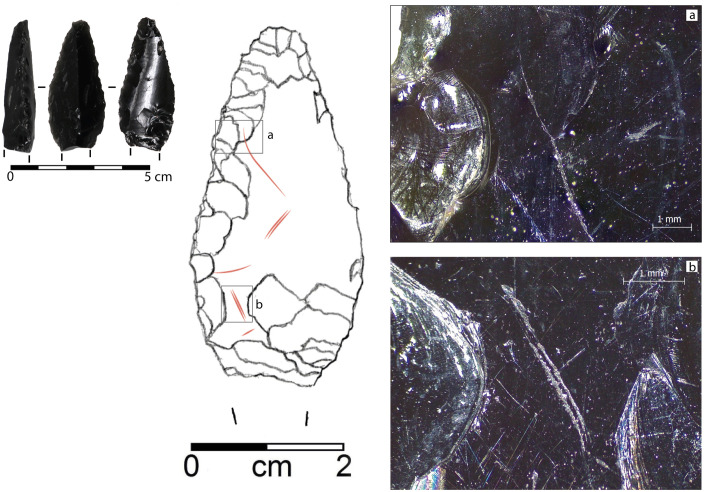
Arrowhead #2 with an alternation of vertical and horizontal incisions on the ventral face.

Arrowhead #2 displays incisions on the ventral surface, alternating between vertical and horizontal lines. The incisions are not centred on the ventral face. Seven incisions were made to create the design. Two of these lines, one near the centre and one towards the proximal end, are doubled (Fig 9B), confirming the deliberate execution of the design. The uppermost incision was made after the inverse retouch, as it overlaps a scar (Fig 9A). This indicates that the incisions were made at least in part after the inverse retouch. The proximal fracture is not diagnostic of projectile use.

**Fig 10:**
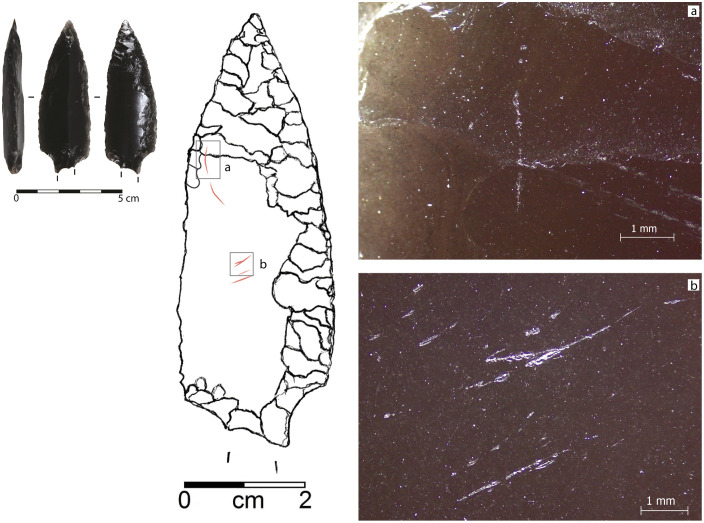
Arrowhead #3 with five incisions on the ventral face, including a group of roughly parallel lines with one doubled incision.

Arrowhead #3 displays five incisions on the ventral face. The incisions lack an obvious structure but include a group of four roughly parallel lines centred in the mesial section. While the configuration may initially appear ambiguous, the presence of a doubled incision within this group (Fig 10B) supports the interpretation of a deliberate and purposeful marking. The uppermost incision overlaps a scar, indicating that at least part of the incisions was made after retouch (Fig 10A). The arrowhead displays a proximal fracture, but it lacks diagnostic features indicative of projectile impact.

**Fig 11:**
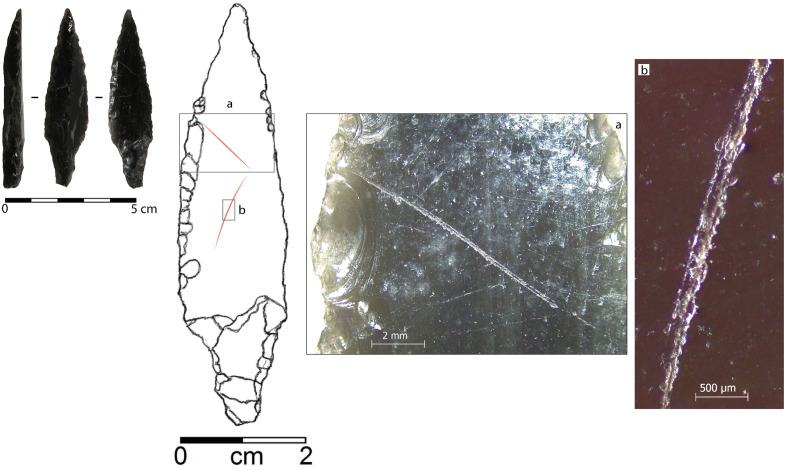
Arrowhead #10 with incisions forming a chevron on the ventral face.

Arrowhead #10 features incisions forming a chevron at the centre of the ventral face. The pattern consists of three incisions, with the lower line intentionally doubled (Fig 11B), reinforcing the intentionality of the design. No direct overlap between the incisions and retouch scars is visible (Fig 11A), suggesting the markings may have been made before, during, or after the retouch process. The arrowhead is complete and shows no diagnostic traces of impact.

**Fig 12:**
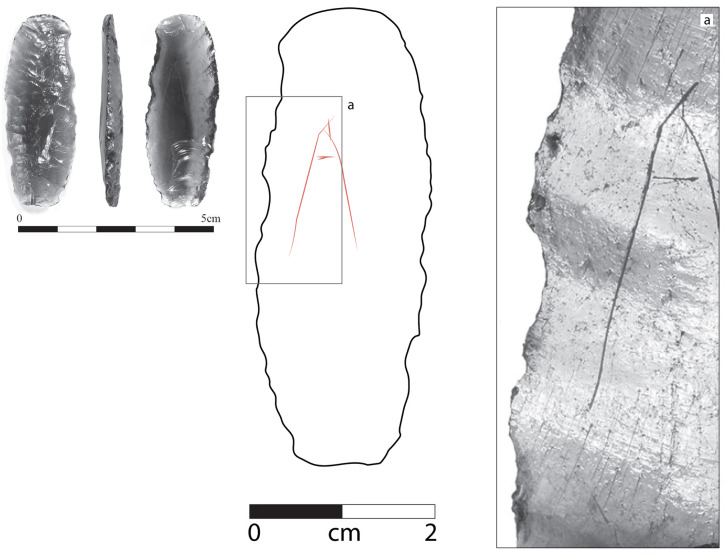
Arrowhead #1 with incisions resembling the letter “A” on the ventral face (pictures are from Balcı 2019, drawing made by the authors).

Arrowhead #1 was previously documented by Semra Balcı in her study of Pre-Pottery Neolithic chipped stone assemblages from Tepecik-Çiftlik [9]. The incisions are positioned in the centre of the ventral face, slightly offset to the left, and loosely form the letter “A”. Based on photographic evidence, it is composed of at least five incisions, although the authors have not examined the artefact directly. The horizontal line forming the “A” shape is visibly doubled (Fig 12A), reinforcing the intentional nature of the incisions. The relative complexity of the incisions also supports a deliberate act of composition. No overlap is visible between the incised lines and retouch scars. The incisions may therefore have been made before, during, or after the shaping of the tool. No visible diagnostic fractures are apparent in the available imagery, however, this arrowhead has not been subjected to a full techno-functional analysis.

**Fig 13:**
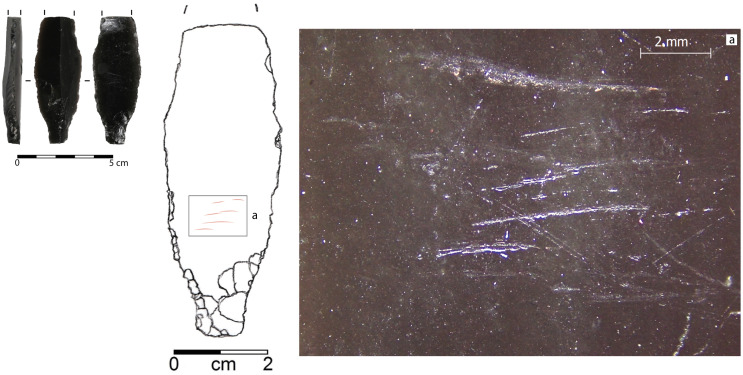
Arrowhead #5 with five parallel incisions on the lower ventral face. The intentionality of the incisions remains uncertain due to lack of repetition and surface disturbance.

Arrowhead #5 displays five parallel incisions on the lower part of the ventral face. Its interpretation remains ambiguous and it is difficult to determine whether the design is intentional or the result of incidental scratching. Although the incisions are clearly visible to the naked eye and show evidence of material removal, they do not meet all three criteria outlined in the methodology section. The lack of repetition and the disturbed nature of the surface (Fig 13A) make it challenging to confidently assess the intentionality of the incisions. Therefore, no functional interpretation can be firmly established. There is no visible overlap between the incised lines and retouch. The incisions may have been made before, during, or after the shaping of the tool. The artefact presents a distal snap fracture that is not diagnostic of projectile use.

**Fig 14:**
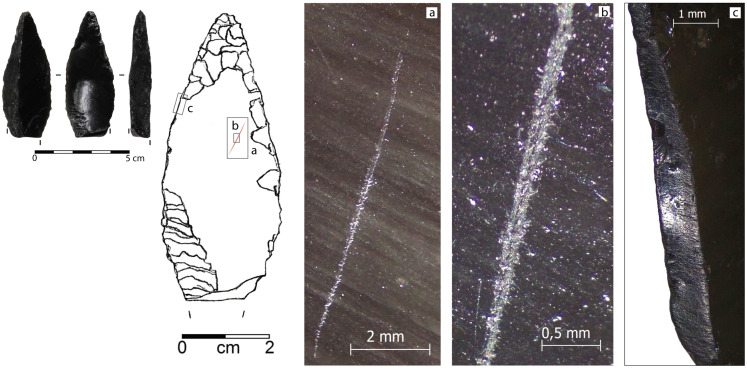
Arrowhead #6 with a single vertical incision on the off-centred ventral face; intentionality uncertain due to lack of repetition.

Arrowhead #6 features a single vertical incision on the ventral face, off-centred. The incision is visible to the naked eye and shows clear removal of material; however, given its isolated nature, its intentionality remains uncertain (Fig 14A-B). Although the surface is well preserved, which could support the hypothesis of a deliberate incision, the lack of repetition of the pattern makes interpretation difficult. In addition to the incision, use-wear traces are observable on the right distal edge. The right edge displays dense, frequent striations perpendicular to the edge. These are wide, long, deep, and have rough bottoms (Fig 14C), which is consistent with a transverse motion such as scraping an abrasive material. There is no visible overlap between the incised line and the retouch. The incision may therefore have been made before, during, or after the shaping of the tool. The arrowhead bears a proximal snap fracture that is not diagnostic of projectile use.

### Synthesis

The twelve incised obsidian arrowheads from Tepecik-Çiftlik display a wide variety of incisions, each differing from one artefact to another. There is no correlation between the characteristics of the incisions and the provenance of the arrowhead within the site (level, structure), the raw material, the manufacture, the typology, or the regularity of the shape or retouch.

None of the incisions appears to be clearly figurative, no design can be unequivocally identified as representing an animal, human figure, plant, or any recognisable subject. Furthermore, no incisions correspond to known decorative or symbolic motifs attested in the broader iconographic corpus of the site, such as those observed on pottery or stamp seals [13]. The incisions observed on the twelve arrowheads from Tepecik-Çiftlik reveal a striking degree of variability in the form and complexity of the lines, as well as in their placement and execution. While every incision is located on the ventral face, their precise positioning varies: some are centred, while others are shifted to one side or dispersed without clear spatial organisation. This uneven distribution suggests that these incisions did not follow a rigid visual convention.

Each group of incisions is unique, with no recurrence across the assemblage. Some designs form shapes such as V-shapes, squares, or crosses, while others seem random. Importantly, none of the incisions can be confidently linked to known symbolic representations from the region. The absence of standardised iconography may imply personal or situational meanings rather than adherence to a broader symbolic system. The complexity of the incisions varies significantly. Some arrowheads bear a single incision or a simple design composed of two or three strokes, while others (such as #4) display more elaborate designs involving up to twenty incisions. However, there is no evident correlation between the complexity of the design and the formal characteristics of the arrowhead. For instance, the most intricate design was not incised on the most symmetrically shaped or finely retouched tool. This lack of correlation suggests that the incisions were not dictated by the tool’s quality or function.

Across the assemblage, the number of incisions ranges from one to twenty, with an average of around eight to nine. Many of these lines were not executed in a single stroke but resulted from two or three roughly aligned passes, rather than careful re-incision of the same line. This practice points to an approach where the visual impact of the incisions mattered more than technical precision.

Regarding the timing of the incisions in relation to the retouch of the arrowheads, no consistent chronology emerges. In three cases, the incisions precede the retouching, as evidenced by incised lines overlapped by subsequent retouch. In three other cases the incisions follow the retouching, as they overlap the retouch. In the remaining six cases, no intersection is visible, leaving the chronological relationship undetermined. This variability implies that incising could occur at different stages in the life of the artefact, possibly reflecting different contexts or motivations.

As for their function, only one artefact (#11) displays a large burin-like fracture suggestive of use in hunting or combat. In most cases, however, the absence of such traces leaves their functional status open. One arrowhead (#6) bears macroscopic traces characteristic of transversal motion on an abrasive material, but the incision on this arrowhead may not have been made intentionally. The incised arrowheads may not all have been intended solely for ballistic use.

This leads to a broader reflection on the role of such incisions in Central Anatolian communities during the Neolithic.

## Discussion

This discussion examines the 12 incised arrowheads from Tepecik-Çiftlik using a multi-scalar approach, focusing on their spatial distribution, provenance, technology, use-wear, and iconography. The data from Tepecik-Çiftlik are compared with similar finds from Central Anatolia. Broader comparisons are then made with extra-regional and ethnographic examples to reassess the significance and function of these rare artifacts.

### Chronological and spatial distribution of incised arrowheads in Central Anatolia

Carter and Milić [3] proposed that incised arrowheads were produced during the Aceramic Neolithic, and that later examples represent either residual materials or objects passed down through generations. However, our observations from Tepecik-Çiftlik, as well as data from other sites, challenge this chronological framework. While incised specimens have indeed been identified in Aceramic contexts, such as Layer 1, Area P at Kömürcü-Kaletepe (8300–8200 BCE), at Sırçalıtepe (mid-8^th^ mil.) and Level 10 at Tepecik-Çiftlik (7100–7000 BCE), they are also clearly attested in contexts associated with the Pottery Neolithic. At Can Hasan III, incised arrowheads were recovered from Phase 2, dated between 7650 and 6600 BCE, at Tepecik-Çiftlik, examples come from Levels 6 to 3, ranging from approximately 6800 to 6100 BCE. These finds provide strong evidence that the practice of incising arrowheads continued throughout the early to late Pottery Neolithic. One specimen from Çatalhöyük was found in a midden dated to the Early Chalcolithic, but due to mixing with topsoil and erosion layers, this context cannot securely support an Early Chalcolithic date for engraved production. Rather than representing a brief or localized phenomenon, the incised points appear to reflect a recurring but infrequent practice embedded in Neolithic lithic traditions across Central Anatolia, a practice that is not attested in neighbouring regions throughout the entire period under consideration. Incised arrowheads are currently known from a limited number of Neolithic sites in Central Anatolia, primarily within the Konya Plain and Cappadocia and are notably absent from important sites occupied during the same period (for example, Aşıklı Höyük [30,31], Musular [9,32], and Köşk Höyük [33]). Despite their broad geographical spread, the distribution of incised arrowheads remains sparse and shows no clear pattern of regional concentration.

### Frequency of incised arrowheads in Central Anatolia

The frequency of incised arrowheads among assemblages remains consistently very low, but significant variations are evident. Considering the two excavated settlements where arrowheads with incisions have been identified, excluding Sırçalıtepe, whose study has only just begun, and Kömürcü-Kaletepe, which is a knapping workshop, significant variations can be observed. The 31 incised arrowheads from Can Hasan III constitute about 3% of the arrowhead corpus (a total of 1,049 arrowheads, [1: 115]). At Çatal Höyük, the 5 incised arrowheads can be compared with the 1,218 arrowheads recorded and studied by T.E. Dogiama in her doctoral thesis [4,34]. They account for only 0.4% of the corpus and are clearly rarer than at Can Hasan III. With about 1.6% incised arrowheads, Tepecik-Çiftlik shows a lower frequency than Can Hasan III, but a significantly higher frequency than Çatal Höyük. Nevertheless, in all cases, this practice remained marginal.

### Finding context of incised arrowheads in Central Anatolia

Almost all of the 54 incised arrowheads recorded from Central Anatolia originate from settlements with exclusively or predominantly domestic occupation. Only four examples come from other contexts: those from Karabatak and Göllüdağ, recovered during surface surveys, are isolated finds unassociated with any known settlement [5,6], while the piece from Kömürcü-Kaletepe was recovered from a knapping workshop located within the obsidian sources of Göllüdağ [7]. Among the examples from villages, the majority unfortunately come from contexts that are either undocumented or poorly recorded. At Tepecik-Çiftlik, none of the arrowheads can be securely linked to a specific structure, and at Can Hasan III they are likewise unassociated with identifiable architectural features. Furthermore, they are not concentrated within specific structures or areas [1]. At Çatal Höyük, two projectiles were recovered from middens in Level South G, one from a midden in Level North H, one from Trench 7 on the West Mound, and one from a floor surface (32492) in Building 160, Level South K [4].

Incised arrowheads never come from particular contexts within the sites concerned, nor do they show any concentration in specific structures or areas. Thus, no specimen is associated with the numerous burials or non-funerary deposits at these sites. They are mixed with the rest of the lithic industry. Conversely, given that such incised arrowheads are already rare within the assemblages in question, their absence from burials and non-funerary deposits cannot be regarded as a distinctive feature.

### Provenance of incised arrowheads in Central Anatolia

Provenance data for incised obsidian arrowheads remain limited. The five examples from Çatalhöyük were identified as originating from Göllü Dağ using PIXE and pXRF analysis [3,4]. At Tepecik-Çiftlik, macroscopic characterisation has shown that six incised points were made from Göllü Dağ obsidian, while six others were sourced from Nenezi Dağ. This confirms that when the obsidian used for incised arrowheads is characterized, it exclusively comes from Cappadocian sources. At these sites, lithic industries are manufactured entirely, or predominantly, from the same obsidian sources, even at locations several hundred kilometers from the outcrops, such as Çatalhöyük and Can Hasan III. The limited geographical spread of these artifacts, restricted to the Konya Plain and Cappadocia, could therefore be partly explained by their reliance on Cappadocian obsidian sources. However, obsidian tools made from the same Cappadocian sources are well-documented across much broader areas, reaching as far as the southern Levant, regions in which no incised arrowheads are found [35–37]. This contrast suggests that the restricted distribution of incised arrowheads is not a result of material availability, but likely reflects specific cultural practices confined to certain Neolithic communities in Central Anatolia with a high frequency of arrowheads.

### Technology of incised arrowheads in Central Anatolia

Based on published data from Can Hasan III, Çatalhöyük, Sırçalıtepe, and Kömürcü-Kaletepe, incised arrowheads show no distinctive technical features in their manufacture compared to other arrowheads recovered from the same sites [1,2,4,7]. Similarly, arrowheads found as isolated discoveries at Karabatak and Göllüdağ display no particular characteristics [5,6]. This is consistent with observations from Tepecik-Çiftlik. Their dimensions and proportions are also identical to those from Tepecik: complete specimens measure on average between 60 and 80 mm in length and approximately 20 mm in width. No clear variation in these criteria is detectable. In most cases, the original blanks cannot be identified; they could be either flakes or blades. Nevertheless, compared to other arrowheads from these sites, they were likely made on blades. At Çatalhöyük, S. Doyle specifies that the five incised arrowheads were indeed made on laminar blanks [4].

The general morphology of these arrowheads varies but remains within the same range as the overall corpus of arrowheads from Central Anatolia, without exhibiting specific traits. The arrowheads are pointed and tanged, often with tangs barely distinguishable from the body of the point. The ventral face is usually unretouched or only lightly retouched; otherwise, no incision would be visible. On the dorsal face, by contrast, pressure retouch is often extensive, covering or nearly covering. The regularity of the retouch and the overall morphology of the arrowheads vary: some show irregular, rather rough retouch with uneven delineation of the edges, while others exhibit parallel retouch and a highly regular morphology.

### Functional analysis of incised arrowheads in Central Anatolia

The use-wear study of the incised arrowheads from Tepecik-Çiftlik provides key insights into how these objects were made and used. The engravings themselves are made with one to twenty strokes, often the incised lines are doubled or tripled, suggesting repeated gestures, possibly to reinforce the design or ensure its visibility. Similar observations can be made from published photographs and drawings of incised arrowheads at other Neolithic sites, indicating a shared technical approach.

Regarding the timing of the incisions relative to the retouching of the arrowheads, no consistent pattern has been identified at Tepecik-Çiftlik, with incisions made before the retouch in three cases and after the retouch in three cases. This suggests that incising may have occurred at different stages of the production process. This contrasts with the clearer pattern observed at Can Hasan III, where Ataman [1] noted that the incisions were systematically executed before the final pressure flaking on the ventral surfaces. The flake scars interrupt the incised lines, indicating that the incision was made prior to finishing. However, based on the illustrations published by Ataman, in at least 23 of the 31 recorded cases the incisions are isolated from the retouch removals. The relative chronology of the incisions in relation to the retouch of the arrowheads can therefore be established for at most eight specimens. Consequently, in our view, it would be premature to conclude that the practice of making incisions prior to retouching was systematic at Can Hasan III.

As for function, the use-wear analysis at Tepecik-Çiftlik shows that only one incised point displays a fracture diagnostic of a projectile impact. This low incidence is consistent with experimental studies, which have demonstrated that diagnostic impact fractures are not systematically produced during use, only a minority of arrowheads develop recognisable breakage patterns, making the presence of only one diagnostic fracture at Tepecik- Çiftlik plausible within a functional use scenario [38–41]. The incised arrowhead from Kömürcü-Kaletepe shows a diagnostic impact fracture [7]. At Can Hasan III, Ataman [1: 266] concluded that the incised arrowheads had indeed been used as projectiles, based on breakage patterns and microscopic wear traces. She argued that these artifacts were indistinguishable in their use-wear from the rest of the projectile assemblage, implying they were not set aside as symbols or ritual items.

Notably, Tepecik-Çiftlik presents a unique case of secondary use among the incised points, but on a point where the incision may not have been made intentionally. While such reuse has been observed for other unincised points from the site [28], this may represent the only known instance of an incised projectile being repurposed after its initial use.

Overall, the functional evidence supports the interpretation that incised points were functionally integrated into Neolithic hunting or armed conflict, rather than being exclusively symbolic or ceremonial.

### Iconography of incised arrowheads in Central Anatolia

**Fig 15:**
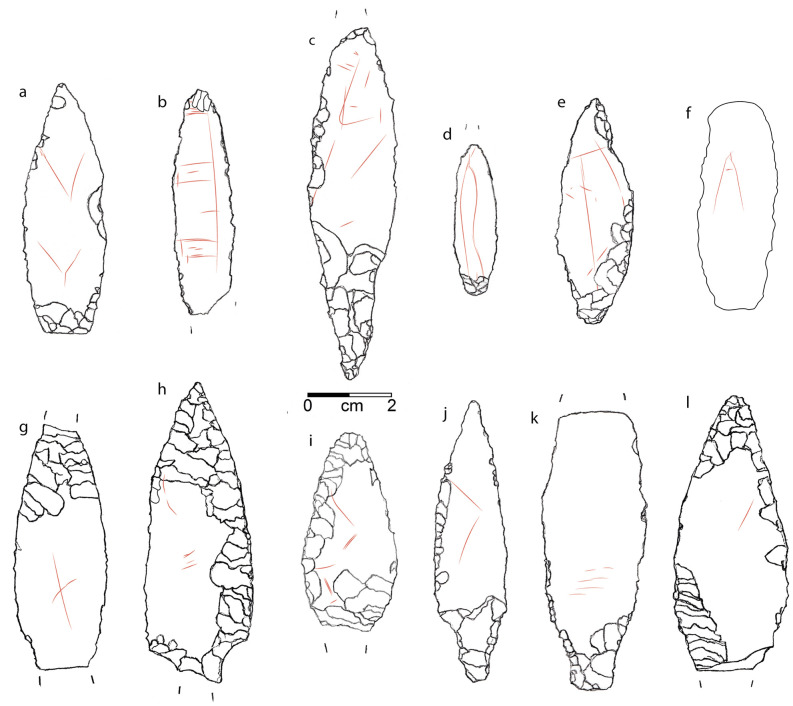
Drawings of the 12 incised arrowheads from Tepecik-Çiftlik.

The iconographic analysis of the incised arrowheads from Tepecik-Çiftlik reveals striking heterogeneity (Fig 15). This diversity echoes earlier observations at Can Hasan III, where Ataman [1] noted that “none of the markings appear to be repeated” among 31 incised specimens. While some motifs at Can Hasan III show partial similarities, such as groups of triangles, none are exact duplicates.

**Fig 16:**
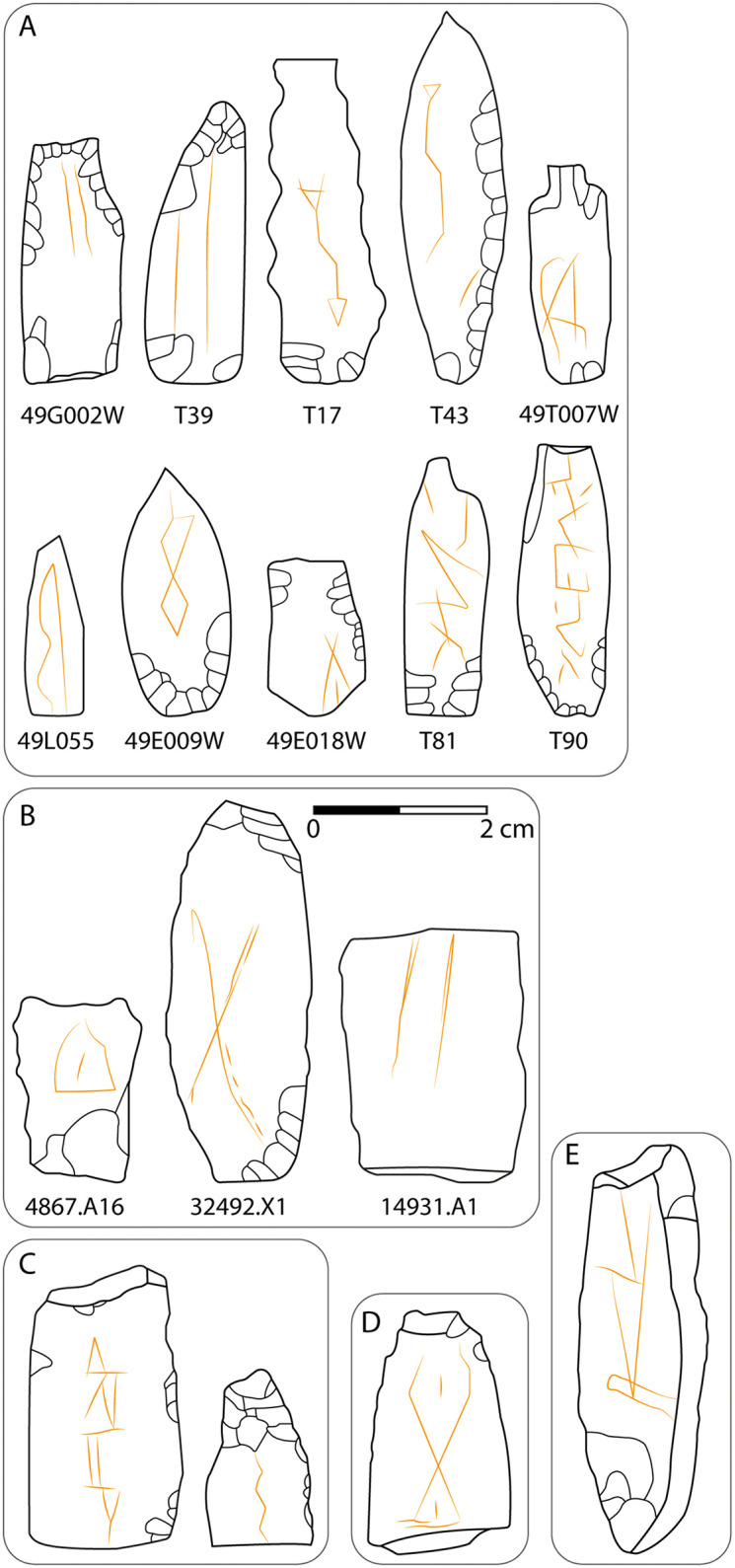
A: Incised arrowheads from Can Hasan redrawn from Ataman 1988; B: incised arrowheads from Çatalhöyük redrawn from Carter & Milic 2013 and Doyle 2021; C: incised arrowheads from Karabatak redrawn from Erdoğu et al. 2007; D: incised arrowheads from Kömürcü-Kaletepe redrawn from Balkan-Atlı and Binder 2000; E: incised arrowheads from Göllüdağ survey redrawn from Balkan-Atlı et al. 2008.

Comparative analysis reveals formal similarities among some incisions across sites. At Tepecik-Çiftlik, a cross (Fig 15G) resembles the design on an incised arrowhead from Çatalhöyük (artifact 32492.X1, Fig 16B). Incisions composed of two wavy lines on another Tepecik- Çiftlik point (Fig 15D) closely match artifact 49L055 from Can Hasan III (Fig 16A). Likewise, the “A”-shaped incision from Tepecik-Çiftlik (Fig 15F) resembles artifact 49T007W from the same site (Fig 16A).

Çatalhöyük also yields arrowheads with incisions comparable to those from Can Hasan III. For example, incised arrowhead 14931.A1 (Fig 16B) displays two vertical parallel lines, similar incisions were found on artifacts T39 and 49G002W from Can Hasan III (Fig 16A). Arrowhead 4867.A16 (Fig 16B) bears a short line inside a triangle, similar to 49E018W from Can Hasan III (Fig 16A).

Additional parallels can be drawn from Karabatak, where two incised points bear a general resemblance to the Can Hasan III assemblage. One features a wavy incision that may depict an incomplete arrow (Fig 16C) comparable to incisions seen on T17 and T43 (Fig 16A). Another exhibits triangular incisions (Fig 16C), reminiscent of artifacts T90 and T81 from Can Hasan III (Fig 16A) and the incised point from the Göllüdağ survey (Fig 16E). Finally, the incised arrowhead from Kömürcü-Kaletepe bears incisions forming a sort of double diamond (Fig 16D), which, although fragmentary, is morphologically similar to 49E009W from Can Hasan III (Fig 16A).

Although no motif recurs in an exact, standardised form, as would be expected from freehand marking on an irregular surface, a set of formal affinities nevertheless emerges across the corpus, consistent with a shared but flexible practice. The presence of comparable incisions suggests they were not entirely idiosyncratic. Rather, they may reflect a commonly understood repertoire of patterns in Neolithic Central Anatolia, reinterpreted according to individual or contextual factors.

### Comparisons with incised arrowheads from other contexts

The fact that, within the chipped stone tool assemblages of Central Anatolia, incisions were made exclusively on projectile points and not on other types of obsidian tools is significant. This specificity raises the question of whether the practice is functionally or symbolically linked to the role of these implements. We therefore sought to identify all known archaeological, historical, and ethnographic cases in which arrowheads bearing incised, engraved, or painted motifs occur. This review is not intended to establish direct analogies, but rather to situate the Anatolian material within a broader comparative framework. Research conducted in the 1980s by K. Ataman provided an excellent starting point [1: 266]. Building on this work, we reassessed previously cited examples and extended the comparison to additional, more distant cases. We excluded all other incised or engraved objects to focus solely on projectile points. This deliberate restriction aims to ensure comparability by concentrating on a single functional category. In doing so, we emphasize a shared functional context, while acknowledging that the comparisons proposed below remain exploratory and limited by the available data.

Arrowheads are extremely abundant and occur in a wide range of chrono-cultural contexts. They have been the subject of numerous specialized studies, particularly regarding their variability and its significance [42–44]. This variability may relate to raw material, morphology, hafting, and fletching. These parameters provide an almost infinite scope for variation and expression. Even when technical and functional constraints are considered, there are countless ways to make an arrow. In this context, the engraved Anatolian points discussed here represent a rare and atypical phenomenon within a very large and otherwise well-documented corpus.

We identified only two cases of comparable incised arrowheads. Both involve slate points, a material that naturally produces plain surfaces when worked and is easy to engrave. In Norway, on the island of Notön, an arrowhead with incised triangles and crosses that J. Goldhann interprets as “entopic and anthropomorphic motifs” was discovered near a waterfall. This point has been compared to slate daggers with engraved decoration found at the site of Nämforsen, dated between 4000 and 1700 cal BCE [45]. Thousands of kilometres from Norway, yet still in the northern hemisphere, K. Ataman mentions and illustrates slate points from Alaska, on Kodiak Island [1: 266, Fig. 90]. These points, from a site dated to the first millennium BCE, bear representations interpreted as symbolic and naturalistic. The archaeological data related to these finds are extremely limited. The number of described specimens is very small, and nothing directly or indirectly illuminates the conditions under which these points were produced or used. Consequently, the analysis of these points cannot provide external insight into the interpretation of the incised Anatolian points.

Given the extremely limited nature of this corpus, we broadened our comparisons to include projectile points made from other materials, particularly bone, ivory, and metal. Although the materials differ, the comparison remains relevant insofar as these objects share the same fundamental practical function, to kill or wound, and follow comparable *chaînes opératoires* to those of knapped points. In all cases, these are shaped implements, with the most elaborately produced specimens requiring hours of labor. Points made from these various materials, stone, bone, ivory, and metal, can, moreover, potentially be resharpened following impact fracture. Below we review all documented cases of figurative motifs on such points.

Projectile points (notably spear points) made of bone and ivory from the European Upper Palaeolithic, associated with hunter-gatherer groups, occasionally bear incised figurative or geometric motifs. However, this should be considered in light of the high frequency of decoration across the full range of material productions in these cultures, especially on objects made from hard animal materials such as bone and ivory. From this perspective, projectile points are not exceptional. In several cases, incisions were made on points, for example at the Aurignacian site of Potočka zijalka in Slovenia [46]. Of the 125 bone points discovered at this domestic site, 34 bear non-figurative incisions, which Camille Jéquier groups into three categories based on the frequency, distribution, length, and depth of the incisions. These points come from the site’s sedimentary layers and are mixed with other points that show no incisions. This context, characterized by a broader and well-documented tradition of ornamentation, differs significantly from the Anatolian case and therefore cannot be considered directly comparable.

Incised points were discovered in the royal tomb PG/789, known as the “King’s Grave,” in the necropolis of Ur in Mesopotamia, within a highly hierarchical society dated between the twenty-sixth and twenty-fourth centuries BCE [47]. These copper-alloy spearheads were found with six bodies grouped at the foot of the dromos. Given their position relative to the burial and the complete military equipment accompanying each individual (helmet, weapon, etc.), the six individuals have been interpreted as the personal guard of the deceased [47: 63, Pl. 189]. The engraved mark visible on the spearhead of each of these presumed soldiers is identical and has been interpreted as a bull’s leg. The same symbol also appears on a metal axe discovered in the same tomb. The bull’s leg is therefore considered a mark identifying the spears as belonging to the guard of the deceased. In other tombs within the same necropolis, a small number of spears also bear engraved marks; for example, one spear displays a highly realistic engraved representation of a cheetah [47: 304, Pl. 190]. This example illustrates a context in which incised arrowheads are standardized and embedded within structured social and symbolic systems, in contrast to the variability observed in the Anatolian material.

In ancient Egypt, from the third millennium BCE onwards, rare incised or engraved motifs appear on various types of objects, including axes, daggers, spears, knives, and chisels. According to W. M. F. Petrie, only three arrowheads bearing incised motifs were known when he published a comprehensive study of Ancient Egyptian artefacts: two in bronze and one in iron [48: 35, 178]. A fourth example, held in the British Museum, is also mentioned by the same author. These rare motifs are not restricted to weapons. The figurative signs, which correspond at least in part to personal names written in hieroglyphic script, also appear on various types of objects and even on the walls of the tombs of the individuals concerned [48: 6]. As in the Mesopotamian case, these practices are associated with literate and hierarchical societies and cannot be directly transposed to the Neolithic Anatolian context.

At Kültepe in Turkey, between 2000 and 1900 BCE, a metal projectile point bearing an inscription is attested [49]. According to J. Tubb, this inscription may represent the name of the craftsman who made it [49]. This interpretation highlights the potential for individual marking practices, although the chronological and cultural distance from the Neolithic assemblage remains considerable.

Phoenician arrowheads from the Early Iron Age, mainly dating between the eleventh and tenth centuries BCE, bear incised inscriptions in the Phoenician alphabet [50,51]. The vast majority originate from poorly documented early finds or from illicit excavations. Of the 73 arrowheads catalogued by M. R. Golub [51], only one comes from a known context, specifically a tomb. The inscriptions correspond to 110 personal names, none of which is repeated. M. R. Golub suggests that these objects may have been used in belomancy (divinatory practices), archery competitions, or warfare, with the engraved name identifying the archer. She acknowledges, however, that no firm evidence supports any of these hypotheses [51: 18]. J. Elayi [50] notes that two of the four inscriptions she studied refer to military functions (“commander,” “chief of a thousand”). Despite their interest, these examples remain difficult to compare directly due to differences in context, chronology, and the presence of writing systems.

Metal arrowheads from south-eastern Arabia, contemporaneous with the Phoenician examples, are characterized mainly by chevron and X-shaped motifs. Several dozen such points have been recorded by Peter Magee [49].

Taken together, these comparative examples highlight the diversity of engraving practices on projectile points but also underscore the absence of close formal or contextual parallels with the Anatolian assemblage, reinforcing the exploratory nature of the present comparison.

Given the absence of close archaeological parallels, we also considered ethnographic cases in a cautious and exploratory manner. The aim is to provide a broader contextual perspective on the possible meanings and functions of incised projectile points. Ethnographic data are not used here as explanatory models, but rather as heuristic tools to outline a range of potential interpretations, while acknowledging the significant chronological, cultural, and technical differences with the Neolithic Anatolian assemblage. Ethnographic cases, from contemporary or sub-contemporary societies, are also very rare and never involve chipped stone points. Among the Eskimo groups of the Bering Strait, for example, harpoons and points made of bone and ivory, as well as other types of objects, bear incisions [12]. In these cases, the incised elements are generally located on the shaft rather than on the active part of the implement, which contrasts with the Anatolian assemblage. According to information collected by E. W. Nelson, these representations are thought to depict the totemic animal of the group to which the hunter belongs and to confer upon the point the qualities attributed to the animal represented [12: 322]. While this interpretation highlights the potential symbolic dimension of incised motifs on hunting equipment, the significant cultural, chronological, and technical differences limit any direct comparison. These data are therefore considered here only as a means of outlining possible interpretative frameworks.

Based on these examples, even when materials other than chipped stone are considered, incisions on projectile points remain extremely marginal on a global scale and across all periods. This observation is not surprising. Engraving a point is not necessarily easy, and the identified cases show that this practice occurs on blanks that readily allow such expression: slate, with its regular surfaces; ivory or bone, which are easy to engrave; or metal, which can be incised or engraved. Chipped stone, with its irregular surfaces resulting from flake scars, is by nature poorly suited to carrying engravings.

Moreover, the cases reviewed differ markedly from those of Central Anatolia in the ninth and eighth millennia BCE. They concern either hunter-gatherer groups (Alaska, Scandinavia, or the Upper Palaeolithic of western Europe) or state-level societies (the royal tomb at Ur and, more broadly, Sout-West Asia from the third millennium BCE onwards). None of the examples identified in the literature involve non-hierarchical agropastoral societies, comparable to those of Central Anatolia. The only common feature shared by all the documented cases is that they represent isolated productions, involving only a very small proportion of arrows within a given region, or even within a single site at a specific and sometimes very brief period.

These highly diverse archaeological cases also reflect very different practices. In hunter-gatherer societies, incised points originate from domestic contexts and do not differ in any clear way from other points. Ethnographic cases rely on testimonies from the groups concerned, which mention incisions made in the context of magical practices, such as enhancing hunting success by schematically depicting on the point an animal whose qualities are thereby appropriated [12: 322]. In state-level societies, the arrowheads discovered in the “King’s Grave” at Ur is the only well-documented case, which is clearly associated with a group of individuals who most likely constituted the personal guard of the deceased. The incisions made on the metal arrowheads, also found on a metal axe from the same tomb, are interpreted as symbols linking these individuals to the deceased. However, alternative or complementary interpretations cannot be excluded, such as a specific symbolic or ritual significance attached to these motifs. For arrowheads bearing Phoenician alphabetic inscriptions, at least the meaning is clear: they record personal names. Specialists of the region consider these to be the names of the owner of the arrowhead or of the chief to whom he was subordinate [51]. Nevertheless, this interpretation rests on no firm evidence, since all but one example derive from undocumented contexts, the sole exception coming from a tomb. Based on these comparisons, we can at least assert that the Central Anatolian points differ from those of the “King’s Grave” at Ur in that they derive from varied contexts that are never funerary or cultic, whereas the Ur examples come from a single tomb and display a single, uniform motif. Moreover, the Anatolian points show heterogeneous incisions, produced over a long period, and come from several sites. We can therefore rule out the possibility that they represent specific productions linked to one or more particular individuals.

Beyond these general observations, these comparisons lead us to exercise caution regarding possible interpretations. Between simple decoration, marks of identification, and magical practices, there is no decisive argument allowing one hypothesis to be distinguished from another; moreover, these interpretations are not necessarily mutually exclusive.

## Conclusions

The analysis of the 12 incised arrowheads from Tepecik-Çiftlik shows that, apart from the incisions, they are indistinguishable from other arrowheads at the site. The incisions themselves are heterogeneous. However, similarities in incisions between sites have been observed, which could potentially indicate a form of shared practice. Tepecik-Çiftlik is indeed comparable to other settlements where such arrowheads have been documented. These sites, however, remain very rare. Given the long duration of this practice, it is clear that it was never widespread, as some Central Anatolian sites partly contemporary with those discussed in this article lack such incised arrowheads.

Nevertheless, this practice is geographically rooted in Central Anatolia, a region where lithic assemblages were predominantly or exclusively made of obsidian, and where pointed-tanged arrowheads long constituted the principal tool type. This tradition never spread beyond the region. This may be explained in part by the fact that pointed arrowheads are a distinctive feature of the region’s sites throughout this extended period. Nevertheless, across the broader PPNA and PPNB cultural sphere, pointed arrowheads are ubiquitous, yet no examples with incisions are known. This indicates that the practice was unique, persisting for more than a millennium solely in Central Anatolia, reflecting significant cultural continuity despite the social and cultural changes evident in material culture and ways of life [44].

The archaeological data relating to incised projectile points from Anatolia do not allow a definite interpretation. The apparently random occurrence of incised points within assemblages, together with the absence of parameters distinguishing them from the rest of the assemblage, does not support ritual interpretations. The variability of the incisions, both within and between sites, suggests that they do not form a standardised shared system of signs either within or across sites. Nevertheless, broad similarities can be observed, which could suggest a shared practice. In this respect, K. Ataman’s hypothesis that these are hunter’s marks, and thus individual markers, remains possible; nevertheless, alternative or complementary interpretations cannot be ruled out.

Comparisons with other incised points from a range of chronological contexts further call for caution. First, no fully comparable cases have been identified in other agropastoral societies. The known examples derive either from hunter-gatherer groups or from state-level societies. Second, even where the archaeological or ethnographic documentation is considerably more abundant, the interpretation of incisions and engravings remains ambiguous. Indeed, some incisions clearly designate individuals (as on Phoenician points) or are associated with individuals (as in the royal tomb of Ur), yet they may potentially refer to different persons: the owner of the point, the leader of the army, or even a deity. Similarly, beliefs possibly associated with the engravings, as in the case of the Eskimos of the Bering Strait, do not exclude other explanations, such as marks corresponding to groups.

The question therefore remains as to why such points are found exclusively in central Anatolia during the 8th and 7th millennia, and neither earlier nor later, nor in neighboring regions. Pragmatic and opportunistic factors should not be overlooked. The sites are characterized by favorable conditions to such practice, a high frequency of arrowheads and the use of obsidian, a stone relatively easy to incise, at least compared to flint. In contexts where arrowheads are less common and/or made from siliceous materials which are much more difficult to incise, the complete absence of incisions is ultimately not surprising. However, these parameters should not obscure the fact that this constitutes a specific practice that cannot be reduced to mere opportunism. Our study demonstrates that, although technically straightforward, the execution of the incisions involves a sequence of gestures (more than twenty in some cases) carried out with specific tools (made of flint, a rare material), and reflects a deliberate intention to mark the point itself, rather than other parts of the arrow that would have been easier to incise, such as the shaft.

At present, it is not possible to go beyond these observations. However, the recent increase in the corpus at Tepecik-Çiftlik, along with the identification of similar arrowheads at other sites, such as Sırçalıtepe [2], provides a broader basis for interpretation. Although the evidence remains limited and the incised motifs are variable, recurrent features (Fig 16) and a coherent spatial and chronological distribution (Fig 1) suggest that these markings may reflect a shared cultural practice. Further discoveries will be necessary to clarify their nature and significance.

## Supporting information

S1 File Inclusivity in global research questionnaire. (PDF)

S2 File Photographs showing the incised patterns with different lightings. a-c: somital lighting of arrowhead #7, b-d: low-angle lighting of arrowhead #9. (TIF)

S3 File Plate illustrating each Tepecik-Çiftlik incised arrowhead. (TIF)
