# Supplementary figures and images for: Intentional incisions on Neolithic Obsidian arrowheads from Central Anatolia: A techno-functional and iconographic approach
Source: PLoS One. 2026 Jul 28;21(7):e0354715. doi: 10.1371/journal.pone.0354715 (PMC13412058; doi:10.1371/journal.pone.0354715)

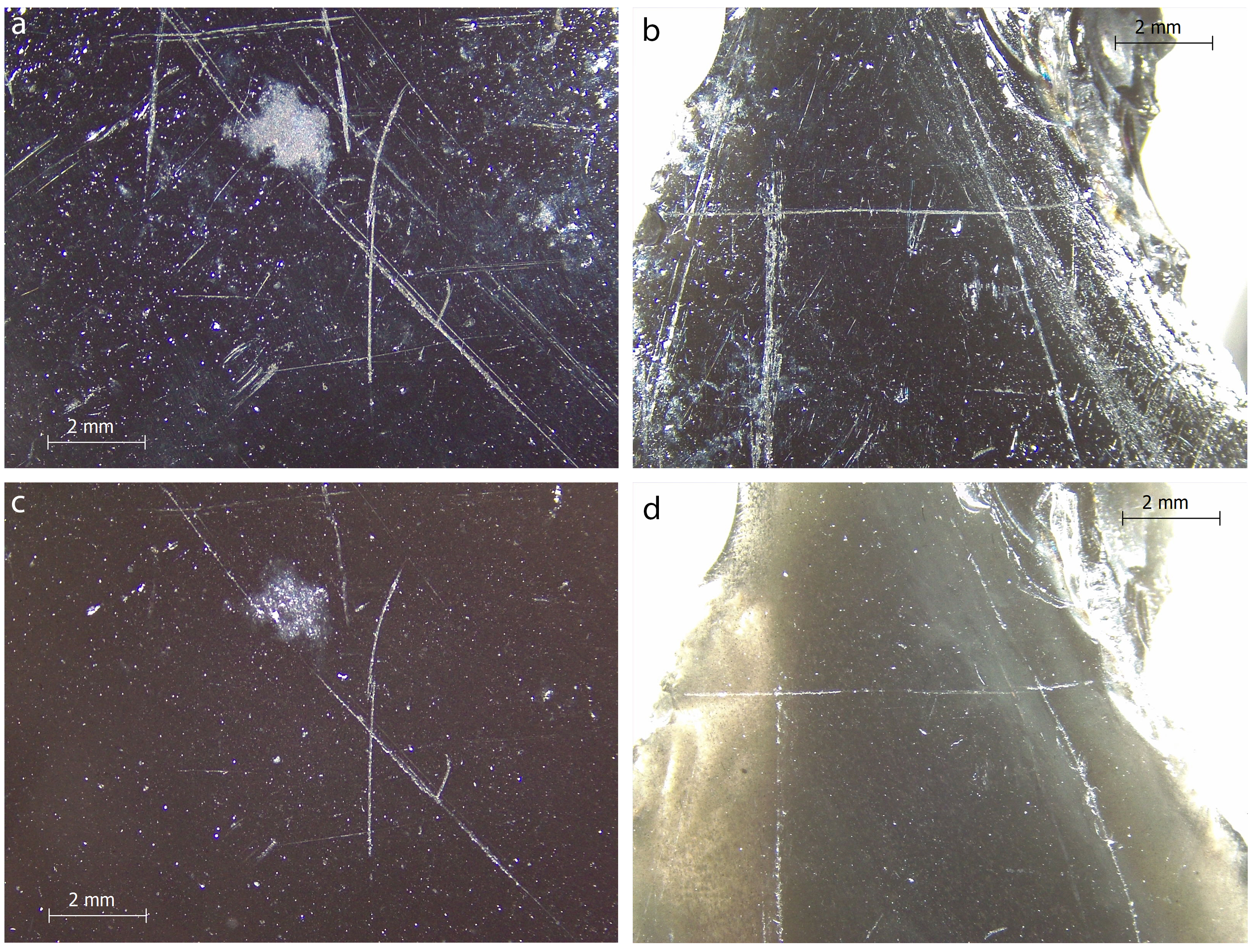

Supplement: S2 File — (TIF) [file pone.0354715.s002.tif]
